# Early renal dysfunction after contrast media administration despite prophylactic hydration

**DOI:** 10.1007/s10554-013-0186-x

**Published:** 2013-02-03

**Authors:** Pawel Burchardt, Przemyslaw Guzik, Piotr Tabaczewski, Tomasz Synowiec, Monika Bogdan, Paula Faner, Anna Chmielarz-Sobocińska, Anna Palasz

**Affiliations:** 1 Division of Cardiology-Intensive Therapy, Poznan University of Medical Sciences, ul. Przybyszewskiego 49, 60-355 Poznan, Poland; 2 Department of Biology and Environmental Sciences, Poznan University of Medical Sciences, Poznan, Poland; 3 Department of General and Vascular Surgery and Angiology, Poznan University of Medical Sciences, Poznan, Poland; 4 Poznan University of Medical Sciences, Poznan, Poland

**Keywords:** Early contrast nephropathy, Coronary angiography, Acute kidney injury, Contrast-induced nephropathy, Serum creatinine

## Abstract

The actual incidence of renal dysfunction after contrast media administration seems to be underestimated, especially in the context of epidemiological data. There are only few data concerning the monitoring of impaired kidney function within a few hours after iodine contrast medium application. Hence, the purpose of this study is to observe the incidence of early renal function deterioration within 12–18 h after administration of iodine contrast media in patients scheduled for elective coronary angiography, who were intravenously and orally hydrated. In addition, the project aims to reclassify the contrast induced nephropathy phenomenon, by identification of early markers of renal dysfunction. Morphology, electrolytes, blood urea nitrogen (BUN), creatinine, low-density lipoprotein cholesterol, triglycerides, high-density lipoprotein, and total cholesterol levels were assessed with the use of typical laboratory techniques in 319 patients referred for coronary angiography. We demonstrated that early deterioration of renal function in patients 12–18 h after administration of contrast during imaging tests (even when appropriate prophylactic hydration was used), may occurred just as an increase (or no change) of serum creatinine level and BUN level and a decrease of creatinine clearance and glomerular filtration rate. Depending on the parameter, the phenomenon can be found in 13–28 % of all respondents. Early renal function impairment defined as above was almost 2 and 2.22 × 10^3^ times (respectively) more frequently observed in our study than contrast induced nephropathy defined by current definitions.

## Introduction

Iodine contrast (JC) media may cause kidney insufficiency [1, 2]. According to the increasing availability of imaging techniques with JC, renal disturbances recently become an important clinical problem. The phenomenon of contrast induced nephropathy (CIN) is currently defined as impairment of renal function which is manifested by an increase of creatinine of 0.5 mg/dL or 25 % from baseline, or a decrease in creatinine clearance of more than 5 mL/min in the period from 24 h to 5 days after administration of contrast agent [3, 4]. Based on the above definition, it occurs in 1–6 % of population undergoing coronary angiography, of which about 0.3 % require dialysis [4, 5]. On the other hand, CIN was observed, even in up to 20 % of patients with severe cardiovascular burden, undergoing imaging tests using JC [5, 6].

The early impairment of renal function within few hours after JC administration has not been clearly defined yet, nor has it been classified. Furthermore, the magnitude of this phenomenon is unknown. The actual incidence of renal dysfunction after JC administration seems to be underestimated, especially in the context of epidemiological data. Hence, the purpose of the project was to observe the incidence of early renal dysfunction within 12–18 h after administration of iodine contrast media in patients scheduled for elective coronary angiography, who were intravenously and orally hydrated. In addition, the project aims to reclassify the CIN phenomenon, by identifying early markers of renal dysfunction.

## Materials and methods

This was a retrospective analysis performed in a single institution in 2010 and 2011. The enrollment period was 16 months. Four hundred and forty two patients were recruited to the study, but due to data deficiency, hydration protocol deviations and exclusion criteria only 319 subjects were joined. From each patient blood samples for laboratory tests were taken twice. For the first time upon on admission to the hospital. A second blood sampling was performed after complete saline administration and within 12–18 h after completion of coronary angiography or percutaneous coronary angioplasty. Patients were periprocedurally (during 24 h) irrigated intravenously (at least 5 h before and up to 10 h after angiography) with commercially available saline enriched with 0.038 g/100 mL of KCL, 0.0394 g/100 mL of (CaCl_2_·6H_2_O), 0.02 g/100 mL, (MgCl_2_·6H_2_O), 0.462 g/100 mL (CH_3_COONa·3H_2_O), 0.09 g/100 mL (C6H5Na3O7·2H2O) (Fresenius Kabi, Poland). The osmolality of media was 301 m OSM/L, The total amount of intravenous liquids were administered according to European Society of Cardiology (ESC) guidelines [7] but were individually modified by physicians (patients with serum creatinine levels above the laboratory norm at admission, received higher volume of saline). Subjects with heart failure were also irrigated according to ESC guidelines [7] and had controlled diuresis. Additionally, our in-ward protocol included 24 h periprocedural (at least 5 h before and up to 10 h after angiography) oral hydration in the amount of 1,500 mL of water for every studied patient. The protocol of irrigation was considered for all patients and only subjects who met these requirements were retrospectively qualified for the study.

Diabetes and hypertension were established according to ESC guidelines [8] or according to previous hospital discharge cards.

### Deterioration of renal function was defined

- as an increase (or no change) of serum creatinine
- decrease (or no change) in creatinine clearance rate (CCR) and glomerular filtration rate (GFR)/expressed by different formulas/.
- decrease creatinine clearance and GFR by more than 5 mL/min and mL/min/1.73 m^2^ respectively.
- decrease in creatinine clearance and GFR by more than 5 %.

Evaluation of creatinine clearance by Cocroft-Gault [9, 10] and GFR by CKD EPI (Chronic Kidney Disease Epidemiology Collaboration) [11] and MDRD (Modification of Diet in Renal Disease) [12] was based on a formula available online (http://en.wikipedia.org/wiki/Renal_function).

#### Inclusion criteria

Study included 319 patients undergoing routine coronary angiography due to the clinical symptoms of ischemic heart disease.

#### Exclusion criteria

Patients with acute coronary syndrome and subjects who had acute coronary syndrome less than 5 weeks earlier were excluded. We additionally excluded patients with heart failure in NYHA IV and or ejection fraction below 30 % (this information was collected from medical records and clinical examination).

The study used two types of angiographic isoosmolar contrast agents: IOMERON 350 (Bracco Imaging, Germany), OPTIRAY 350 (Tyco Healthcare, Germany).

The maximum contrast dose was calculated according to proposed [13] formula: (5 times the weight of the patient/baseline serum creatinine level). The contrast index’ (amount of contrast used, to a maximum dose of contrast) as well as ratio of used contrast per creatinine clearance [14] were assessed.

### Laboratory parameters

Creatinine levels, were assessed by using buffered kinetic Jaffe reaction without deproteinisation kit-C system Cobas 6000 (Roche Diagnostics, Germany). Method was calibrated by isotope dilution mass spectrometry). Blood urea nitrogen (BUN), uric acid, triglicerydes (TG), cholesterol, high density lipoproteins were quantitatively determined by enzymatic colorimetric method using Cobas 6000 (Roche Diagnostics, Germany) system with sophisticated reagents. Low density lipoproteins (LDL) were measured by indirected way with Freidewald formula. TSH was measured by electrochemiluminescent method with using the Cobas 6000 (Roche Diagnostics, Germany) analyzer with Roche reagents Germany. Electrolytes, were measured by ion selective, potentiometric method using Cobas 6000 (Roche Diagnostics, Germany). Morphology was assessed using Sysmex XT2000i (Sysmex, USA).

#### Statistics

Normality was tested in the Shapiro–Wilk’s W test. At normal distribution of variables the T-Student test for two independent and dependent variables was used. Mann–Whitney test for two independent variables and Sign test as well as Wilcoxon matched pairs test for two dependent variables were used at abnormal variables distribution. The binomial test were used for comparing standard and ‘non standard’ definition of CIN.

The results are given as mean ± SD. The statistical significance was established when *p* < 0.05. Statistical analysis was conducted using STATISTICA 8.0 software.

## Results

The study was conducted in 319 patients undergoing elective coronary angiography at the age of 60.62 years ± 8.63. The mean body mass index (BMI) in the study population was 29.09 ± 4.97 kg/m^2^. Fifty nine percent of patients were male, 41 % women. Hypertension, impaired glucose metabolism (glucose intolerance, impaired fasting glucose and diabetes mellitus combined), previous myocardial infarction, previous renal insufficiency (GFR/by MDRD/<60 mL/min/1.73 m^2^) and heart failure (up to NYHA III) subsequently were reported in 85, 44.5, 37.3, 14.7 and 19.1 %, respectively. Fifty six percent of patients who underwent elective coronarography, had percutaneous transluminal coronary angioplasty (PTCA) undertaken within the same procedure. The evaluation of examined laboratory parameters are shown in Table 1. Patients received an average of 112.46 ± 57.85 mL of contrast (coro + PTCA) and 0.54 ± 0.54 Gy of radiation (coro + PTCA). The procedure lasted a total of 26.19 ± 20.86 (coro + PTCA) minutes, and total fluoroscopy time was 5.5 ± 7.47 min (coro + PTCA). Patients were periprocedurally hydrated intravenously with commercially available K^+^, MG^2+^ and Ca^2+^ enriched saline solution in an amount of 1,614.42 ± 221.3 mL and 1,500 mL of fluid po (per os). The maximum contrast dose (5 times the weight of the patient/baseline serum creatinine level) was 514.58 ± 155.46 mL. ‘The contrast index’ (amount of contrast used, to a maximum dose of contrast) was 0.24 ± 0.17. Ratio of used contrast per creatinine clearance [10] was equal to 1.29 ± 1.02. Seventy nine point one percent of patients were taking angiotensin converting enzyme-inhibitors (ACE-i), 9.7 % of patients were taking angiotensin receptor blockers (ARB), 85.9 % of patients were taking beta blockers (BB), diuretics and statins were taken respectively by 39.22 and 95.01 % of studied subjects. Study groups (with or without renal deterioration) did not differ in scope of pharmacotherapy profiles.

**Table 1:** Characteristics of studied group

	Studied group Mean ± SD (N = 319)
Age (years)	60.6 ± 8.6
BMI (kg/m^2^)	29.1 ± 4.97
Hypertension (%)	85
Glucose metabolism disturbances (IFG, IGT, DM) (%)	44.5
Heart failure (%)	19.1
Previous myocardial infarction (%)	37.1
Previous PTCA (%)	36.04
Previous CABG (%)	3.1
Dyslipidemia	38.1
Chronic kidney disease (%) (GFR < 60 (mL/min/1.73 m^2^)	14.7
Creatinine baseline (mg/dL)	0.93 ± 0.46
Creatinine after 12–18 h from JC administration (mg/dL)	0.87 ± 0.47
RBC baseline (×10^6^/μL)	4.57 ± 0.47
RBC after 12–18 h from JC administration (×10^6^/μL)	4.3 ± 0.5
HGB baseline (g/dL)	13.9 ± 1.4
HGB after 12–18 h from JC administration (g/dL)	13.04 ± 1.54
HCT baseline (%)	40.55 ± 3.71
HCT after 12–18 h from JC administration (%)	38.4 ± 4.31
BUN baseline (mg/dL)	37.0 ± 12.44
BUN after 12–18 h from JC administration (mg/dL)	29.65 ± 10.67
Uric acid (mg/dL)	5.67 ± 1.63
TSH (μU/mL)	1.8 ± 1.43
TC (mg/dL)	178.94 ± 68.4

*BUN* blood urea nitrogen, *CABG* coronary artery by-pass graft, *GFR* glomerular filtration rate, *HCT* hematocrit, *HGB* hemoglobin, *JC* iodine contrast, *LDL* low density lipoprotein, *PTCA* percutaneous transluminal coronary angioplasty, *RBC* red blood cells, *TC* total cholesterol, *TSH* thyroid stimulating hormone

### Assessment of renal function after 12–18 h after administration of JC, and hydration compared to measurements on admission to the hospital

Statistically significant decrease in creatinine (0.93 ± 0.46 vs. 0.87 ± 0.47 mg/dL, *p* < 0.001) and BUN (37 ± 12.4 vs. 29.64 ± 10.67 mg/dL, *p* < 0.001), higher creatinine clearance (100.33 ± 36.2 vs. 107, 67 ± 38.5 mL/min, *p* < 0.001) and glomerular filtration rate according to the MDRD formula (81.6 ± 23.03 vs. 88, 75 ± 25.39 mL/min/1.73 m^2^, *p* < 0.001) or CKD EPI (83.1 ± 19.7 vs. 87.2 ± 18.8 mL/min/1.73 m^2^ *p* < 0.001) were noticed in control parameters assessment in whole group (after the JC and the use of prophylactic hydration). In addition, we observed statistically significant decrease in the number of red blood cells (×10^6^/μL) (4.56 ± 0.47 vs. 4.3 ± 0.51, *p* < 0.001, hemoglobin (HGB) (g/dL) (13.9 ± 1.4 vs. 13.0 ± 1.53, *p* < 0.001), hematocrit (HCT) (%) (40.5 ± 3.7 vs. 38.4 ± 4.3, *p* < 0.001).

### Markers of impaired renal function

**Fig. 1:**
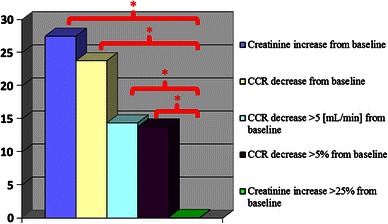
Comparison of various CIN criteria in studied population. *Legend*: *p < 0.05
B. Reduction (or no change) of CKD according to EPI, reduction of CKD according to EPI by more than 5 mL/min/1.73 m^2^ and a decrease of CKD according to EPI by more than 5 % were found in 28.5, 11.2, and 11.9 %, respectively.
C. Reduction (or no change) of MDRD, MDRD decrease by more than 5 mL/min/1.73 m^2^ and a decrease in MDRD by more than 5 % was found in 23.8, 14.7 and 15.9 % of respondents respectively.
D. After 12–18 h of contrast administration, an increase or no change in the serum creatinine and BUN levels (despite of hydration) were observed in 27.6 and 12.8 % of respondents respectively, an increase in creatinine of >25 % occurred in 0.09 % of all patients.

A. Decrease (or no change) in creatinine clearance, decrease in creatinine clearance by more than 5 mL/min and decrease in creatinine clearance by more than 5 % were found in 23.8, 14.4, and 13.7 % of respondents respectively (Fig. 1).

Individuals with (no change or) reduction in creatinine clearance or glomerular filtration rate (established according to CKD EPI or MDRD), with decrease of these parameters for 5 mL/min/1.73 m^2^ and their decline by more than 5 %, significantly differed from the rest of the patients by: age, the amount of contrast media used and presences of combined glucose metabolism disturbances (IFG, IGT, DM) (Table 2). In male versus female group there were significantly higher rate of (no change or) reduction in CCR (17.24 vs. 6.9 %, *p* = 0,01) or GFR (CKD EPI 22 vs. 6.9 %, *p* < 0.001 or MDRD 16.9 vs. 6.9 %, *p* = 0,014). Although, statistically insignificant trend in male group was observed where patients show a decrease of these parameters for 5 mL/min/1.73 m^2^ and their decline by more than 5 %. Men had also significantly more often increased creatinine after angiography (19 vs. 9 % *p* = 0.04). The percent number of patients who suffered from heart failure or chronic kidney disease at the beginning of the study were comparable between patients with or without impaired renal function after contrast media administration.

**Table 2:** Parameters which significantly differ the groups of patients with various definition of post-contrast renal deterioration phenomenon (the asterisk shown on the second row mean that all comparisons within the 3 groups are statistically significant)

	CCR decrease >5 (mL/min)		CCR decrease >5 %		CCR decrease	
	YES/N = 46	NO*/N = 273	YES/N = 44	NO**/N = 275	YES/N = 76	NO***/N = 243
Age (years)	57.5 ± 8.6	61.1 ± 8.5			58.47 ± 8.5	61.1 ± 8.5
Creatinine (baseline) (mg/dL)	0.8 ± 0.2	0.95 ± 0.5	0.93 ± 0.8	0.93 ± 0.38	0.9 ± 0.6	0.96 ± 0.5
Creatinine (after 12–18 h from JC administration) (mg/dL)	0.9 ± 0.2	0.86 ± 0.5	1.05 ± 0.9	0.84 ± 0.33	0.96 ± 0.7	0.86 ± 0.5
CR (after 12–18 h from JC administration) minus CR(baseline) (mg/dL)	0.3 ± 1.4	−0.09 ± 0.1	0.3 ± 1.4	−0.09 ± 0.1	0.2 ± 1.1	−0.1 ± 0.1
Ratio of CR (after 12–18 h from JC administration)/CR (baseline)	1.1 ± 0.06	0.9 ± 0.08	1.1 ± 0.06	0.9 ± 0.08	1.08 ± 0.07	0.9 ± 0.08
RBC (baseline) (×10^6^/μL)	4.7 ± 0.5	4.5 ± 0.5	4.7 ± 0.5	4.5 ± 0.5	4.7 ± 0.5	4.5 ± 0.5
RBC (after 12–18 h from JC administration) (×10^6^/μL)	4.5 ± 0.6	4.3 ± 0.5	4.5 ± 0.6	4.3 ± 0.5	4.5 ± 0.55	4.3 ± 0.5
HGB (baseline) (g/dL)	14.5 ± 1.2	13.8 ± 1.4	14.4 ± 1.3	13.8 ± 1.4	14.4 ± 1.3	13.8 ± 1.4
HGB (after 12–18 h from JC administration) (g/dL)	13.7 ± 1.4	12.9 ± 1.5	13.65 ± 1.4	12.9 ± 1.5	13.6 ± 1.5	12.9 ± 1.5
Ratio of HGB (after 12–18 h from JC administration)/HGB (baseline)					0.95 ± 0.05	0.9 ± 0.06
HCT (baseline) (%)	41.9 ± 3.5	40.3 ± 3.7	41.7 ± 3.5	40.4 ± 3.7	41.65 ± 3.6	40.3 ± 3.7
HCT (after 12–18 h from JC administration) (%)	40.2 ± 4	38.1 ± 4.3	40.1 ± 4	38.1 ± 4.3	40.2 ± 4.2	38.1 ± 4.3
HCT (after 12–18 h from JC administration) minus HCT (baseline) (%)			−1.5 ± 2.3	−2.24 ± 2.6	−1.5 ± 2.45	−2.2 ± 2.6
Ratio of HCT (after 12–18 h from JC administration)/HCT (baseline)			1 ± 0.05	0.9 ± 0.06	1 ± 0.06	0.9 ± 0.06
BUN (baseline) (mg/dL)					33.9 ± 9.4	37.6 ± 13
BUN (after 12–18 h from JC administration) minus BUN (baseline) (mg/dL)	−4.52 ± 6	−7.8 ± 6.7	−3.77 ± 5.9	−7.9 ± 6.7	−4.3 ± 6.6	−7.8 ± 6.7
Ratio of BUN (after 12–18 h from JC administration)/BUN (baseline)	0.88 ± 0.2	0.8 ± 0.15	0.9 ± 0.17	0.8 ± 0.15	0.88 ± 0.2	0.8 ± 0.15
TC (mg/dL)	211.5 ± 146	173.5 ± 41.5				
LDL (mg/dL)	114.7 ± 48.4	96.8 ± 32.3				
Ratio of Contrast volume/SCR (baseline)	1.02 ± 0.7	1.3 ± 1.05			1.0 ± 0.64	1.4 ± 1.08

*BUN* blood urea nitrogen, *CR* serum creatinine, *CCR* creatinine clearance, *HCT* hematocrit, *HGB* hemoglobin, *JC* iodine contrast, *LDL* low density lipoprotein, *RBC* red blood cells, *TC* total cholesterol

* *p* < 0.05 between patients with CCR decrease >5 (mL/min) and without

** *p* < 0.05 between patients with CCR decrease >5 % and without

*** *p* < 0.05 between patients with no change or CCR decrease versus CCR increase

### Differences between the standard definition of CIN and the proposed new definition

Standard definitions of contrast induced nephropathy were considered: (A) when GFR or CCR decreased by 5 mL/min/1.73 m^2^ or 5 mL/min, (B) creatinine level increases by 25 % or 0.5 mg/dL. The significant differences were found between the standard definition A and CIN interpreted as a decline in (GFR), *p* < 0.001 (by CKD EPI), and *p* < 0.001 (by MDRD) or creatinine clearance *p* = 0.0026.

We found statistically significant differences between the standard definition B, and CIN interpreted as an increase (or no change) in serum creatinine level, *p* < 0.001. Renal function impairment defined as a decrease (or no change) in GFR, CCR or increase (and no change) in serum creatinine level was almost 2 and 2.22 × 10^3^ times (respectively) more frequently observed than nephropathy defined as A or B.

### The association between disparity of creatinine clearance (calculated according to Cocroft-Gault formula) and serum creatinine level

**Fig. 2:**
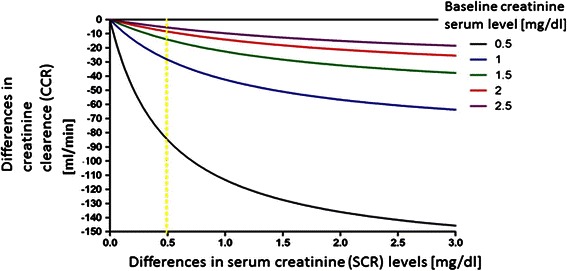
Non-linear association between SCR and CCR according to various baseline creatinine levels

The association between disparity of creatinine clearance and disparity of pure creatinine serum levels is non linear and is shown at Fig. 2. The simulation was performed for a woman, 175 cm, 80 kg, 50 years, with different baseline serum creatinine levels (from 0.5 to 2.5 mg/dL).

## Discussion

Contrast induced nephropathy has ischemic etiology. JC probably causes reduction of oxygen tension in both medulla and cortex thus increasing the oxygen tension imbalance between these two compartments. Ischemia is intensified by an increased release of endothelin and adenosine [15, 16] and may be enhanced by the production of reactive oxygen species [3]. One can not exclude a direct toxic effect of contrast on renal tubular epithelium as well [17–19]. Despite many attempts to prevent CIN, the best results were obtained with hydration of the patients during the periprocedure period [3, 7].

In clinical practice, the phenomenon of contrast nephropathy is described as impaired renal function from 24 h to 5 days after administration of contrast agent. However, in defining CIN phenomenon, still large discrepancies exist. These relate to the time after administration of contrast, the impairment of renal function, the choice of parameters used to describe renal function and extent of their changes.

In our study we noticed improvement of the baseline renal function after 12–18 h from contrast use and after the hydration of the patients (3,150 mL during the period immediately before and after the procedure/1,500 mL orally + intravenously 1,614.42 ± 221.3 mL/). However, in absolute levels the creatinine increased (or no changed) in 28 % of subjects. Twenty eight point seven percent and 27.7 % of patients subsequently decline in creatinine clearance, or (variously defined), glomerular filtration rate. It should be noted, that individuals in whom we have noticed this phenomenon were older and had significantly higher baseline and control levels of: BUN, creatinine, HGB, RBC and HCT. Additionally those patients more often than others experienced combined glucose metabolism disturbances. Surprisingly, the percent number of patients who suffered from heart failure or chronic kidney disease at the beginning of the study was not significant and didn’t differ between patients with or without impaired renal function after contrast media administration. It is probably due to the fact that we included only those subjects who displayed up to moderate heart failure. However, we observed nonsignificant statistical trend toward more frequent number of subjects with the baseline chronic kidney disease, which is consistent with the literature [4–6, 14]. The renal dysfunction was more often observed in male group. Only possible explanation for this is the fact, that male group was more frequently (although statistically insignificant) affected by heart failure and chronic kidney disease before study, what may have interfered with their renal pattern, during our analysis.

The decrease in creatinine clearance of 5 mL/min, was the most radical definition of CIN we have found in the literature [3, 4], and it was observed in approximately 14 % of all analyzed subjects. In our study any impairment of renal function according to our criteria was noticed twice as often as when defined by standard definition. On the other hand, when we considered the CIN creatinine increase of 25 % or about 0.5 mg of the output [3, 4], the phenomenon has occurred in only 0.9 % of the studied population. Although the deterioration in renal function manifested by an increase (or no change) of creatinine was up to 2.22 × 10^3^ times more frequently observed then current CIN definition.

The use of different parameters to the interpretation of CIN is not random in our study. Serum creatinine levels is not a equivalent of creatinine clearance, which is in clinical conditions mostly calculated according to Cocroft-Gault formula. This is confirmed by the simulation performed for a woman, 175 cm, 80 kg, 50 years, with different baseline serum creatinine levels (from 0.5 to 2.5 mg/dL). We showed, that the association between disparity of creatinine clearance (between measurements at the beginning and at the end point) and disparity of pure creatinine serum levels (between measurements at the beginning and at the end point) is non-linear, and depends on the creatinine baseline (Fig. 2). Thus, the higher the level, at the baseline the decline in creatinine clearance is greater.

These data (as well as 3 other [20–22]) therefore seem to confirm the need to revise the criteria for the diagnosis of CIN, particularly on the basis of early (within several hours) [22] measurements of creatinine, creatinine clearance and glomerular filtration rate. This is an extremely significant clinical problem, especially in the context of discharging the patients (undergoing routine coronary angiography) from hospital on the next day after the procedure. This is also important due to the fact that CIN worsens the prognosis of patients, being an independent risk factor for future chronic kidney disease. The proposed interpretation of contrast nephropathy phenomenon based on the early decline in renal function after administration of JC, therefore allows to separate high risk groups as well as to have time to implement appropriate clinical management. We propose in such cases to extend the hospitalization time until the return of serum creatinine to the baseline levels. Additional saline and acetylcysteine irrigation is then to be individually considered and the creatinine and BUN levels are to be strictly controlled. After discharge every patient is to be obligatory scheduled for ambulatory nephrological inspection.

Limitation of the study is the fact that this is a retrospective, observational analysis. Hence, there was no single control time measurement of the analyzed parameters. Patients were irrigated periprocedurally according to ESC guidelines and it took 24 h. However, according to ward protocol we used to start irrigation at least 5 h before procedure, and in other cases (when time of starting the angiography was late) we continued it up to 10 h after the procedure. It means that patients who received the same volume of hydration may differ each other in volume of irrigated liquids provided (in period) before as well as after the procedure, what depends on the time of starting the procedure. Furthermore, the patients’ weight on the next day after irrigation is unknown. In addition, we do not know the levels of BUN and creatinine, in the period from 3 to 7 days after administration of contrast medium. Creatinine assessment method error is ±8 %, but every indication was performed under identical conditions, so the ‘constant error’ was eliminated. Another limitation of this study was that two similar but not identical contrast media of similar osmolality and iodine content were used. The undeniable strengths of our analysis include the fact of homogeneity of the study population according to pharmacotherapy, and homogeneity of hydration procedures.

Giving the implications of this study, perhaps larger prospective study should be considered in the future.

## Conclusions

Impairment of renal function 12–18 h after contrast agent administration (even when prophylactic hydration is used), may result in no change or an increase of creatinine and BUN and a decrease (or no change) in CCR and GFR. Depending on the parameter, the phenomenon was detected in 13 to 28 % of our patients. The early renal dysfunction identified as above is twice more frequent than the most stringent current definition of CIN. The results need confirmation in a large clinical group.
